# EDCs and Estrogen Receptor Activity: A Pathway to Safer Chemical Design?

**DOI:** 10.1289/ehp.122-A339

**Published:** 2014-12-01

**Authors:** Julia R. Barrett

**Affiliations:** Julia R. Barrett, MS, ELS, a Madison, WI–based science writer and editor, is a member of the National Association of Science Writers and the Board of Editors in the Life Sciences.

Estrogen receptors are some of the primary targets of endocrine-disrupting chemicals (EDCs). In a new report in this issue of *EHP,* biochemical, structural, biophysical, and cell-based experiments reveal critical information about the activity of 12 EDCs at the molecular and atomic levels.^1^ The EDCs tested, including the plasticizer bisphenol A and the flame retardant tetrachlorobisphenol A, are suspected to have a role in the development of various cancers and developmental, reproductive, and metabolic disorders via interactions with estrogen receptors.

**Figure:**
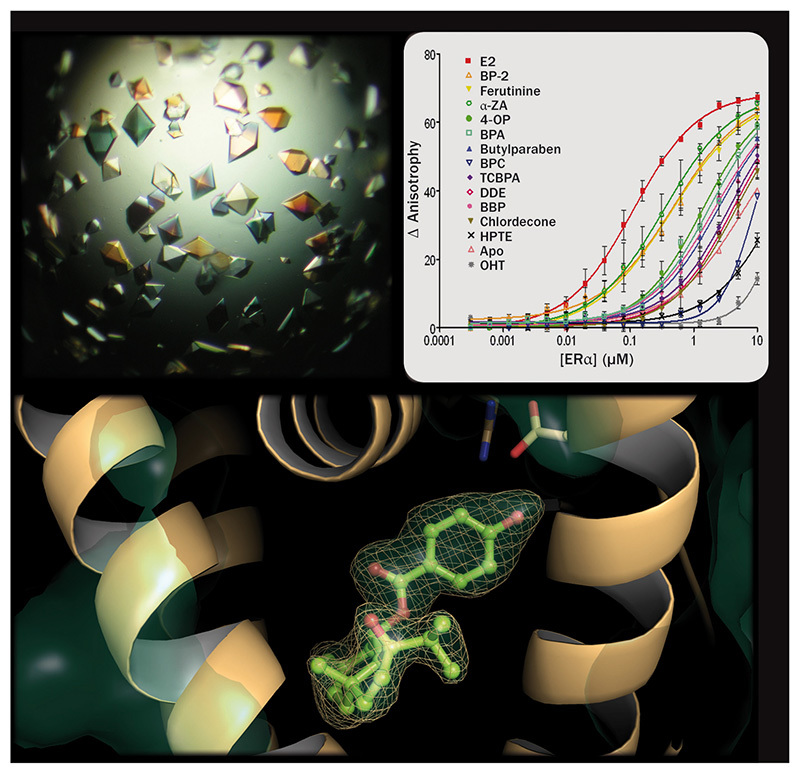
A new study provides the visualization, at atomic resolution, of the estrogen receptor bound to various endocrine-disrupting chemicals. The insights gained may help guide the development of safer chemicals. © Vanessa Delfosse and William Bourguet

“A better understanding of the many ways by which environmental pollutants interfere with nuclear receptor signaling will help in predicting the residual hormonal activity of an existing industrial compound and rationalizing the development of new analogues devoid of nuclear receptor activities,” says study coauthor William Bourguet, team leader at the Center for Structural Biochemistry, Montpellier University, France. EDCs can undermine the endocrine system by either mimicking or blocking (antagonizing) endogenous hormones, or by interfering with their synthesis, metabolism, or transport.^2^^,^^3^

Estrogen receptors, which occur as ERα and ERβ subtypes, have been particularly well studied with regard to EDCs.^4^^,^^5^ The predominant subtype of estrogen receptor varies by tissue throughout the body, although the two are sometimes co-expressed. The ligand that normally interacts with these receptors is 17β-estradiol, which is critical to the growth and development of tissues throughout the body. Interference with the action of 17β-estradiol has been associated with cancer, infertility, obesity, and diabetes.^6^^,^^7^

Predicting the potential outcomes of EDC exposure is complicated by the fact that numerous unrelated chemicals can bind to ERα and/or ERβ.^1^^,^^8^ “Because the chemical structures of environmental compounds are generally very different from those of natural compounds, their binding modes are difficult to predict,” says Bourguet.

In the current study, the researchers parsed estrogen receptor activity based on a detailed examination of functional domains characteristic of both receptor subtypes: the N-terminal domain, which includes activation function 1 (AF-1), and the C-terminal ligand-binding domain, which contains AF-2. AF-2 activity depends on a ligand being bound, but AF-1 activity is independent of the ligand. AF-1 integrates signals from AF-2 and other pathways to modulate the receptor’s ultimate activity—gene transcription.

“Although not directly linked, the two activation functions can work synergistically or independently depending on the nature of the bound ligand,” says Bourguet. “One of our aims was to evaluate the role of each function in both receptor subtypes and in the presence of the different compounds,” he says.

To this end, the researchers first generated HeLa cells containing a reporter gene paired with wild-type ERα or ERβ or with mutant receptors lacking AF-1. Subsequent assays revealed which of the 12 EDCs could activate each subtype as well as how critical AF-1 was to the activation process.

All the EDCs could bind to the receptors; however, some activated ERα and ERβ equally, while others activated only one or the other. The presence of AF-1 modulated the activity, particularly for ERβ, whereas AF-2 was more important for ERα activation. Competitive assays demonstrated the EDCs’ binding affinities relative to 17β-estradiol, which ranged from very similar to 50,000-fold lower values. Next, protein crystallization experiments demonstrated each chemical’s fit in the ligand-binding pocket of ERα. These structural data reflected the findings from the *in vitro* assays and helped explain the compounds’ differential activity on ERα and ERβ.^1^

The precise response of an estrogen receptor to a specific ligand hinges on the cellular context and the presence of cofactors,^9^ and it’s still unknown whether *in vitro* observations reflect what happens *in vivo*. “This is a critical aspect to understanding how estrogenic chemicals really behave in tissues, and why this study is important,” says Yukitomo Arao, staff scientist in the NIEHS Reproductive and Developmental Biology Laboratory. “In general, it is important to understand the differential tissue functionality of various estrogenic compounds; more narrowly, for example, AF-1 activity is regulated by the gene promoter context and is cell-type specific,” says Arao, who was not involved in the study.

A potential weakness of this study is the use of a constitutive active mutant of ERα to facilitate crystallography, which Arao suggests might alter the chemical–receptor interactions. Another potential weakness is the exclusive use of HeLa cells; AF-1 varies between cell types and tissues, so its behavior in one cell line may not reflect how it will act in another. “However, the authors mentioned the differential estrogenic activity of compounds in different cells in their discussion,” Arao says, “and I hope they will make similar stable cell lines using different cell types to reevaluate EDCs’ estrogenic activities.”
